# Determination of Microplastics in Omega-3 Oil Supplements

**DOI:** 10.3390/foods13101434

**Published:** 2024-05-07

**Authors:** Moonhae Kim, Juyang Kim, Seulah Park, Dowoon Kim, Jaehak Jung, Dongha Cho

**Affiliations:** 1 Department of Bio-Health Convergence, College of Biomedical Science, Kangwon National University, Chuncheon 24341, Republic of Korea; kmhsmsystech@gmail.com; 2 Korea Institute of Analytical Science and Technology, Seoul 04790, Republic of Korea; kjy@kiast.co.kr (J.K.); psa@kiast.co.kr (S.P.); kdw@kiast.co.kr (D.K.)

**Keywords:** microplastics, omega-3 supplements, health functional foods, μRaman

## Abstract

Microplastics (MPs) are ubiquitous in the environment, posing a threat to ecosystems and causing increasing concerns regarding their impacts on the human body through exposure. However, there has been limited research on the presence of MPs in functional foods, despite them being consumed for health improvement. This study aimed to investigate MP occurrence in various omega-3 oils and oil products in the Korean market and its relation to the source of raw material or manufacture. MPs were investigated in omega-3 capsules and raw oil, sourced from both plant-based (PB) and animal-based (AB) sources. We developed a method of direct filtration with acetone washing for collecting and characterizing MPs larger than 5 μm using micro-Raman spectroscopy. The average number of MPs by mass was found to be 1.2 ± 1.7 MPs/g for PB raw oil, 2.2 ± 1.7 MPs/g for AB raw oil, 3.5 ± 3.9 MPs/g for PB capsule oil, and 10.6 ± 8.9 MPs/g for AB capsule oil. Polypropylene and polyethylene terephthalate were the major MP species (83–95%) found in omega-3 oil. The proportions based on size range remained consistent across all groups, with a trend of being detected at higher rates as the size decreased. The results reveal that the main reason for the MP contamination of omega-3 oil is not the source of raw material but the manufacturing and packaging process.

## 1. Introduction

Plastics are irreplaceable in modern life due to their versatility and wide range of applications. Over the past two decades, global plastic production has exploded from 234 million tons (Mt) in 2000 to 460 Mt in 2019 [1]. With the increase in plastic usage, plastic litter has become a significant threat to the environment. Furthermore, since the emergence of the term, ‘microplastics’ (MPs), which are less than 5 mm in size [2,3], they have been recognized as a ubiquitous material and a novel kind of pollutant of ecosystems [4,5]. Recent studies on the occurrence of MPs in environmental media have shown that MP pollution is widespread, with MPs found in the ocean [6,7], fresh water [8,9], air [10], and soil and sediments [11,12]. Due to plastics having the property of being highly resistant to degradation, MPs accumulate through the food chain and are consequently consumed by humans [13]. Especially in the case of seafood, the bioaccumulation of MPs in the marine web may cause a problem for food safety from the perspective of the food chain [14]. As the exposure of the human body to MPs is becoming an increasing concern, analysis has focused on foods such as beverages [15], milk [16], seafood [17], fruits and vegetables [18], and edible oils [19,20]. However, data regarding their occurrence in food sources and harmonized identification methods are still lacking, indicating that more data and research are needed [21].

Omega-3 fatty acids, such as docosahexaenoic acid (DHA) and eicosapentaenoic acid (EPA), are polyunsaturated fatty acids characterized by a carbon double bond in the omega position of their methyl terminal end. On account of their health benefits to humans, they have become one of the most widely consumed dietary supplements. The global omega-3 market size accounted for USD 7.5 billion in 2022 and is projected to reach around USD 15.1 billion by the end of 2032 [22]. Omega-3 supplement oils are mainly made from fish oils extracted from blue-backed fish such as herring and mackerel, but consumer interest in plant-based ingredients made from algal oil for vegetarians and vegans has grown in recent times. Since the source of the omega-3 supplements is fish oil, there have been concerns that omega-3 supplements may contain MPs due to their contamination and accumulation in marine organisms used for the raw material. However, to date, there have not been no reports evaluating the presence of MPs in omega-3 oils [23]. In this context, it is necessary to determine the current level of MP contamination in omega-3 supplements.

Internationally standardized methods have been developed for determining MPs in water matrices by ISO/TC 147/SC 2/JWG 1, but not yet in food or oil matrices. The standard methods for identifying and quantifying MPs utilize vibrational spectroscopic techniques coupled with microscopes, such as micro-Fourier transform infrared spectroscopy (μFTIR) and micro-Raman spectroscopy (μRaman) [24]. Using these methods, MPs can be identified through comparing the collected spectra with those in databases. Visual images of MPs, containing information about their size and shape, can also be obtained. Due to spectroscopic and time limitations, a practical cut-off size of 20 μm is applied for μFTIR and 5 μm for μRaman to analyze the entire filtrated area either via automated mapping or individual particle analysis. Considering the potentially low concentration of MPs in oil, the cut-off size of 5 μm was chosen for μRaman in this study to achieve the highest possible detection limit.

In this study, we aim to investigate, for the first time, the presence of MPs in various omega-3 supplements available in the Korean market. We collected encapsulated omega-3 oil samples, which represent the form of the product typically taken by consumers, as well as the raw oil itself, stored in non-plastic bottles before encapsulation and provided by the manufacturer. Furthermore, these samples were categorized as being from animal-based (AB) or plant-based (PB) sources to examine whether there are potential differences in the presence of MPs related to the oil source. In addition to simple analysis of MPs in omega-3 oil, the detection trends of MPs among the categorized sample groups were compared and statistically analyzed. To this end, we developed a simple method to analyze MPs in omega-3 oil, including a simple recovery test, and rigorously controlled environmental factors to minimize possibility of MP contamination in the laboratory. To the best of our knowledge, the present study is the first study that investigates the presence of MPs in omega-3 oils using μRaman. This study can contribute to fill the knowledge gap of not only the MPs contained in health functional food but also the source of MPs in omega-3 oils.

## 2. Materials and Methods

### 2.1. Preparation of Omega-3 Oil Samples

Commercial encapsulated omega-3 oil samples sourced from the Korean market, as well as raw material samples which are not encapsulated, provided directly from the manufacturer, were collected. Due to confidentiality concerns, the commercial brand names are not disclosed. A total of 14 raw omega-3 oils and 21 encapsulated omega-3 oil products were included. The raw oils and products were categorized into PB oils and AB oils based on their sources. The numbers of samples by sample type and oil source are listed in Table 1.

**Figure 1:**
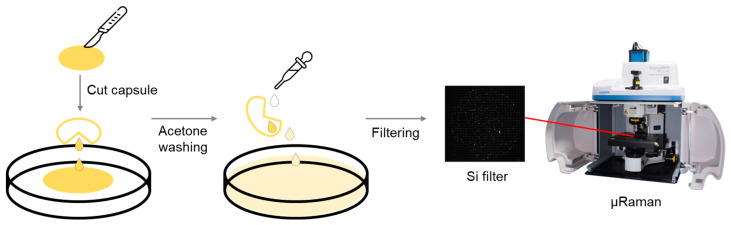
A schematic diagram of the analytical procedure of capsule oil sample.

A schematic diagram of the overall procedure for sample preparation, particularly for capsule oil samples, is shown in Figure 1. Capsule oil samples were carefully cut using a metal scalpel, and the oil inside was poured into a pre-cleaned Petri dish. The sample mass was determined based on the manufacturer’s recommended serving size. This was calculated by subtracting the amount corresponding to the capsule from the total amount for the product (capsule + oil). Since the amount of oil in one serving capsule typically ranges from 2 g to 3 g, 2.5 g of raw oil was used for the raw oil samples.

The cut capsules were rinsed with pre-filtered acetone (Samchun Pure Chemicals, Pyungtaek, Republic of Korea) dispensed using a pasture pipette to collect the remaining oil in the capsule. The mixture of omega-3 oil sample and acetone was then poured into a filtration system and filtered directly through a 5 μm silicon filter (10 mm × 10 mm square, SmartMembranes, Halle, Germany). Any remaining oil on the walls of the Petri dish and the filtration funnel was washed off with acetone. A total volume of 30 mL of acetone was used, and sampling was conducted quickly to minimize any possible damage caused by acetone.

### 2.2. Determination of Microplastics Using μRaman

Raman analysis was performed using an XploRA Plus confocal Raman microscope (Horiba, France) to detect microplastics. The Raman system and detailed parameters were the same as in our previous study [25]. Briefly, a 532 nm laser and a 1024 × 256 pixel-cooled charge-coupled device (CCD) detector were used with 10% laser power. The spectral range was set to 1020–3400 cm^−1^ using a 1200 groove/mm grating, and the exposure time was set to 1 × 2 s. Mosaic images and collected spectra were processed using the Particlefinder^TM^ module in Labspec 6 software. All spectra were screened for plastics using the classical least square algorithm (CLS). Spectra of polyethylene (PE), polypropylene (PP), polystyrene (PS), polyvinylchloride (PVC), polyurethane (PU), polyamide (PA), poly(ethylene terephthalate) (PET) and poly(methyl methacrylate) (PMMA) were set as standard references for the CLS fitting. Each measured spectrum was calculated as the sum of all the references and the theoretical composition. The results of the CLS spectra were manually inspected and adjusted if necessary to avoid missing data or false positives using the KnowitAll^TM^ spectrum matching software with a Raman library. Only results with a minimum Hit Quality Index (HQI) of 70% were classified as MPs.

### 2.3. Cross-Check for Possible Misidentification of PE Using μFTIR

μFTIR analysis was performed using FTIR spectroscopy on a microscope (LUMOS II, Bruker Optics, MA, USA) equipped with a 32 × 32 pixel focal plane array (FPA) detector. IR images were acquired in transmission mode at a spectral resolution of 12 cm^−1^ within a spectral range from 3800 to 900 cm^−1^, utilizing one scan time. μFTIR was specifically employed to distinguish particles that might be erroneously identified as PE using Raman spectroscopy.

### 2.4. Environmental Control to Prevent MP Contamination

To prevent microplastic contamination, only glass and non-plastic materials were used during the sampling and filtering process. All the sample preparation, pretreatment, and filtration steps were conducted inside a laminar flow bench equipped with a ULPA H14 filter (Sinan science industry, HSCV-1300, Gwangju, Republic of Korea) within the laboratory to mitigate contamination from indoor airborne microplastics. Solutions, including ultrapure water and chemical reagents, were pre-filtered using a 0.7 µm GF/F filter (Whatman, Buckinghamshire, UK) and then a 1 μm stainless steel filter (KIAST, Seoul, Republic of Korea) prior to use. Glassware was cleaned with filtered ultrapure water before laboratory experiments. To prevent any contamination, samples were covered with aluminum foil before moving outside the laminar flow bench. Nitrile gloves and cotton coats were worn during all processing steps to minimize the risk of contamination.

### 2.5. Statistical Analysis

Descriptive statistics were used to analyze the significance between the sample groups. The mean and standard deviation (SD) were used to describe continuous variables. The unpaired *t*-test was used to analyze differences in MPs abundance of different groups. All the statistical analysis were performed using Microsoft Excel 2019.

## 3. Results

### 3.1. Preliminary Tests with Simple Recovery Test

Omega-3 oil itself is a clear solution and can be directly filtrated through a silicon filter for analysis using μRaman or μFTIR. However, since its vibrational spectrum also corresponds to the range for μRaman and μFTIR analyses, it is desirable to thoroughly remove omega-3 oil from the filter surface. Furthermore, after pouring the oil inside the capsule onto the Petri dish, the remaining oil inside the capsule, as well as the Petri dish and filtration funnel, must be cleaned with a solvent. Initially, hot water containing non-ionic surfactant was utilized as a washing solvent. However, the main issue with using a water-based solution was that the capsule easily dissolved in water, making sampling difficult. Additionally, it was difficult to remove oil both on the filter surface and also on laboratory equipment such as the Petri dish and filtration funnel. For these reasons, we tested using acetone as an alternative cleansing solution.

Acetone may cause damage to some types of plastics, such as PS and styrene copolymer, but is less likely to affect PP, PE, and PET. A recent study reported a simplified method for extraction of MPs using acetone [26], meaning that MPs have tolerance to acetone. To evaluate the potential damage that may occur during the pretreatment, PS microspheres (65 μm, Spherotech, IL, USA) were added to the Petri dish, followed by pretreatment. After the treatment, surface morphology was compared using a Scanning Electron Microscope (SEM, JEOL JSM 7001F, Tokyo, Japan). SEM images of PS microspheres before and after acetone treatment are shown in Figure S1. There was no notable size change or damage on the surface of the acetone-treated sample, but slightly dissolved traces could be seen at the bottom of the PS. Considering that the major determinant species of MPs are PE and PP, acetone appears to be a good choice for removing oil due to its amphipathic characteristics. We aimed to minimize the sampling time as much as possible to reduce the potential damage to polymers. The total sampling time per sample was less than 10 min, including filtration and washing steps, meaning that MPs could be exposed to acetone for the same duration.

A simple recovery test was conducted using spherical PE MPs with a size range from 75 μm to 80 μm (Cospheric, CA, USA). The PE spheres were added to a slide glass and manually counted using μRaman. The PE spheres on the glass were transferred into the Petri dish after washing the glass with acetone. Raw oil was pre-sampled on the Petri dish before adding spheres. The PE spheres with oil were filtered, and the number of spheres on the filter were counted. Initially, 118 PE spheres were added, and 117 spheres were counted on the filter (Figure S2). Thus, the calculated recovery rate of microplastics in oil was over 99%.

### 3.2. Procedural Blank Test and Limit of detection (LOD)

The LOD was determined to establish the threshold for the number of MPs resulting from contamination following the ‘best practice guidelines’ outlined by Schymanski et al. [27] and also defined in ISO/DIS 16094-2 [24] as the reporting limit (RL). This value is used to define the limit for which the laboratory can provide a quantitative result. In this study, a particle size of 5 μm was applied as the lower limit due to the filter pore size and the instrumental limit for μRaman in automatic mode. It is recommended that 10 blank samples are analyzed for determining LOD. The LOD was determined as follows:

*LOD (MPs) = mean_blanks_* + 3 × *s_blanks_* (1)

where *mean_blanks_* is the average number of MPs identified in the procedural blanks and *s_blanks_* is the standard deviation.

In this study, 12 procedural blank samples were analyzed. Procedural blank samples were prepared in the same manner as for omega-3 oil sampling, but they did not containomega-3 oil. The obtained value was 0.3 ± 0.5 and the calculated LOD was 1.8 MPs, based on the tests in this study. This LOD demonstrates the cleanliness of the studied environmental area, indicating that the contamination from airborne particles or apparatus is very low, allowing for the determination of MPs down to a minimum size of 5 μm in oil.

### 3.3. Determination of Microplastics in Omega-3 Oil Samples

**Figure 2:**
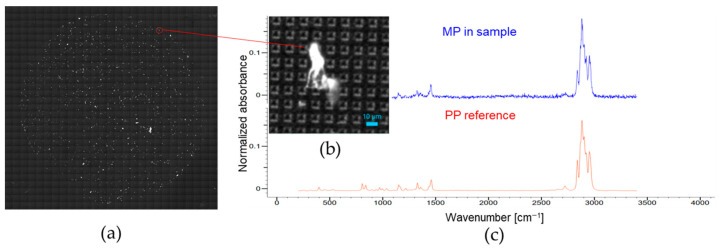
Image of (**a**) mosaic image; (**b**) detail image of an identified MP; (**c**) comparison between acquired and reference spectra).

**Figure 3:**
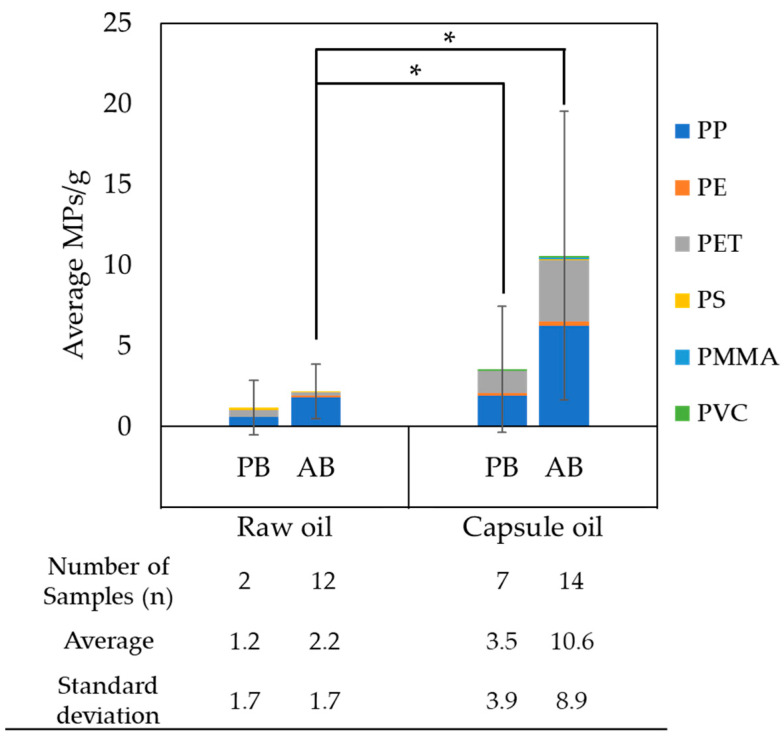
Average numbers of MPs (MPs/g) with standard deviation and polymer composition of each omega-3 sample group (plant-based (PB) and animal-based (AB)). Unpaired *t*-test, * *p* < 0.05.

The determination of MPs was performed using a pre-tested method for omega-3 oil samples. An example of a mosaic image of an Si filter and detected particles, along with their spectra, is depicted in Figure 2. The average number of MPs and the standard deviation for each sample group are shown in Figure 3. Individual results of the numbers of MPs in omega-3 sample are shown in Table S1.

For comparison between groups, the results are presented as the average number of MPs per gram of sample, with individual data normalized accordingly. The average number of MPs per gram was 1.2 ± 1.7 MPs/g for PB raw oil and 2.2 ± 1.7 MPs/g for AB raw oil. When considering the raw oil alone, there was no significant difference in the abundance of MPs between the two sources (*p* > 0.05). During oil processing, filtration and centrifugation steps were applied to refine and clean the oil. Typically, oil undergoes multi-step cleaning, including filtration at 100 μm and 0.5 to 1 μm. Therefore, it is noteworthy that the source of the raw oil has no effect on MP pollution in terms of MP ingestion.

The average numbers of MPs per gram for PB capsule oil and AB capsule oil were 3.5 ± 3.9 MPs/g and 10.6 ± 8.9 MPs/g, respectively, around 3 to 5 times greater than in raw oil. There was significant difference in the abundance of MPs between the two sources (*p* < 0.05). Additionally, the standard deviation for each sample group is high compared to the average value, indicating that MP contamination is dependent on the cleanliness of the manufacturing process, including encapsulation. Therefore, it can be inferred that the relatively lower number of MPs found on average in PB capsule oil may be attributed to the fact that PB oil, representing a more recent development, is likely produced in facilities with inherently cleaner environments in terms of MPs.

**Figure 4:**
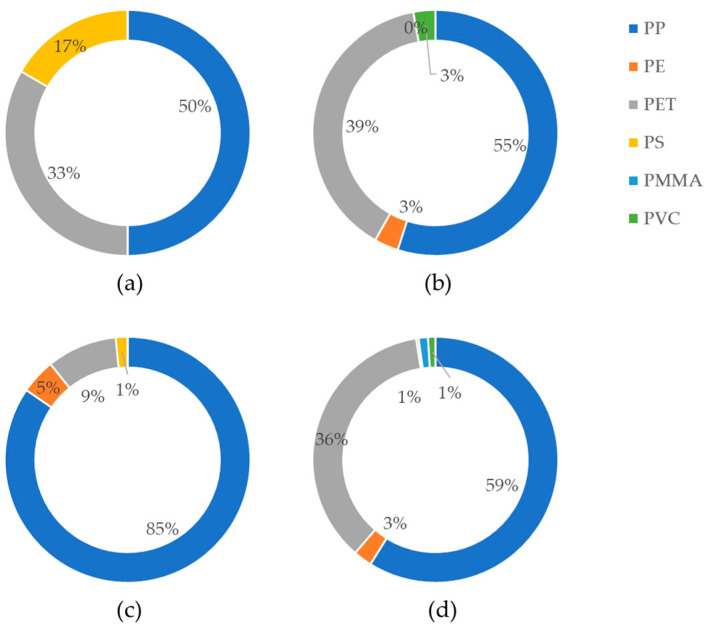
Distribution by polymer composition of (**a**) plant-based (PB) raw oil; (**b**) plant-based (PB) capsule oil; (**c**) animal-based (AB) raw oil; (**d**) animal-based (AB) capsule oil.

As depicted in Figure 3 and Figure 4, PP is the most abundant species, accounting for more than 50% of the total MPs across all groups. In addition, PET was found in both capsule oil sample groups, accounting for more than 36% and thus representing a large portion of the detected MPs. PP and PET were the predominant species in omega-3 oils (83–95%). This indicates that PET can easily become contaminated during the encapsulation process. Moreover, considering the higher amount of PP in the capsule oil, PP is also the major source of MP contamination during the encapsulation process.

On the other hand, there was no significant difference in the abundance of MPs between the raw and capsule PB oils (*p* > 0.05), but there was for AB oils (*p* < 0.05). These results clearly show that the main reason for MP occurrence in the final product is not the oil itself but rather the encapsulation process, at least for AB oils. Further research is necessary to confirm that MP contamination is from the encapsulation process because there is no direct match between capsule oils and their corresponding raw oil given the different sample numbers of each group. However, as a result, it can be concluded that final products consumed by consumers may contain MPs, regardless of how clean the raw material is. There may be concerns regarding experimental aspects, namely, that the different sampling methods for raw oil and capsule oil can lead to differences. However, the possibility of contamination from the laboratory was at least checked by investigations using procedural blank tests. The occurrence of MPs from the packaging or the packaging environment is a common and anticipated problem. Thus, it could be more important to control the packaging-derived contamination to avoid MP exposure through food intake.

**Figure 5:**
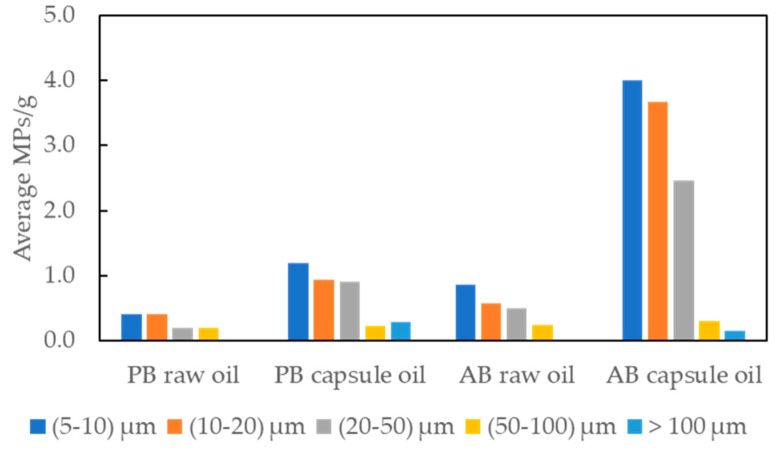
Distribution by particle size of the identified MPs in omega-3 oil samples (plant-based (PB) and animal-based (AB)).

As can be seen in Figure 5 and Figure S3, there is a trend of increasing numbers of MPs detected as the size range decreases. The distribution according to size classification appears consistent across all groups, with the most dominant size for all sample groups being the range of 5 to 10 μm, comprising 33% to 40% of the total. Notably, the size range of 5 to 20 μm accounted for 60% to even 73%. Considering that the practical size limit for μFTIR is 20 μm, these results reveal the efficacy of μRaman for analyzing MPs in omega-3 oil. MPs smaller than 5 μm were not considered in this study due to technical limitations and time constraints. However, it is implied that MPs of smaller size may be more prevalent in the samples, necessitating further investigation and the enhancement of technical and analytical capability in the future.

### 3.4. Identification of PE Spectra and Cross Analysis Using μFTIR

**Figure 6:**
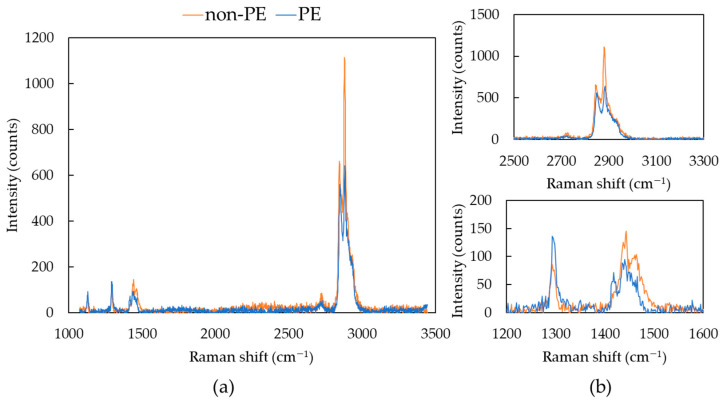
Raman spectra of reference PE and non-PE particles found in omega-3 oil samples: (**a**) full measured spectrum range; (**b**) enlarged spectrum range.

As mentioned in the under-developed test method standard ISO/DIS 16094-2, the Raman spectrum of PE is very similar to that of hydrocarbon compounds such as oil, wax, and stearates, requiring the problem of false positives to be considered [24]. Analyzing the entire filter area is time consuming, so we allocated 2 s for individual particle analysis. The total analysis time was at least 5 to 6 h for all particles greater than 5 μm on the filter. Thus, there is a limitation with regard to obtaining clearer spectra of individual particles with a high signal-to-noise ratio. In some samples, some spectra that could easily be mistaken for PE were observed, which appeared not to be from PE (expressed as ‘non-PE’). Raman spectra for reference PE and non-PE particles are shown in Figure 6. Signals at wavenumbers characteristic of PE in Raman spectroscopy were observed at 1127 cm^−1^ for C-C stretching, 1293 cm^−1^ for CH_2_ twisting, three bands at 1417 cm^−1^, 1439 cm^−1^, and 1465 cm^−1^ for CH_2_ bending, and two bands at 2849 cm^−1^ and 2883 cm^−1^ for C-H stretching [28,29]. The difference in Raman spectra between non-PE and PE can be seen at several points. First, the intensity of CH_2_ bending at 1417 cm^−1^ is low, almost resembling two bands rather than three bands. Second, the intensity at 1293 cm^−1^ is lower than the three bands between 1417 and 1465 cm^−1^. This difference can also be observed in low-molecular-weight hydrocarbons such as oil or wax. Moreover, the intensity of the shoulder at 2960 cm^−1^ is higher and of a comparable ratio to the intensity of the shoulder at 2883 cm^−1^.

**Figure 7:**
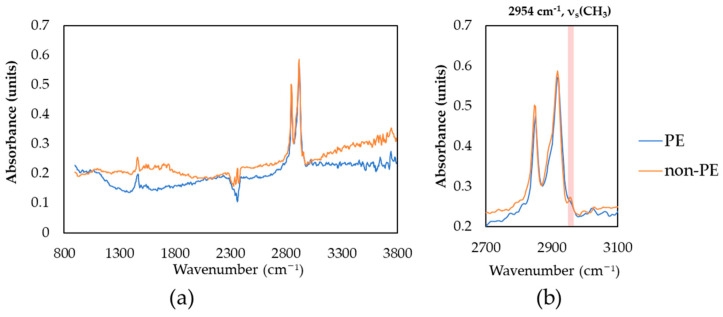
FTIR spectra of reference PE and non-PE particles found in omega-3 oil samples: (**a**) full measured spectrum range; (**b**) enlarged spectrum range.

These samples were analyzed using μFTIR to confirm whether or not the particles were PE. The FTIR spectra are shown in Figure 7. Both spectra are quite similar, and even more concerning, the fingerprint region is not clear due to the low signal-to-noise ratio. In the FTIR spectra, bands characteristic of CH_2_ stretching can be observed at 2843 cm^−1^ and 2913 cm^−1^. A difference can be seen at the 2954 cm^−1^ shoulder, which is clearly divided and also can be seen in Raman spectra. This shoulder can be assigned to CH_3_ stretching, which can appear at the methyl group of the end of the carbon chain. Ideally, PE has a very low portion of methyl ends, but the portion increases in low molecular hydrocarbon, including oil, stearates, and lipids [30]. On the basis of the combined results of μFTIR and μRaman, this spectrum is excluded from the MP results. Clearer spectra for identification were obtained for other MPs such as PP, PET, and PS, in contrast to PE, so IR cross-checking was not applied.

## 4. Conclusions

To our best knowledge, this is the first study to demonstrate the presence of MPs in commercial omega-3 supplement oil. Raw and capsule oils were sorted based on their sources as PB or AB. A simple filtration method employing acetone washing was applied, achieving around a 99% recovery rate for PE microspheres. μRaman was employed for the determination of MPs greater than 5 μm, and μFTIR was used for cross-checking the PE spectra. Rigorous environmental controls were maintained within the laboratory, and the calculated LOD from twelve procedural blanks was 1.8 MPs. The results revealed an MP concentration of 1.2 ± 1.7 MPs/g for PB raw oil, 2.2 ± 1.7 MPs/g for AB raw oil, 3.5 ± 3.9 MPs/g for PB capsule oil, and 10.6 ± 8.9 MPs/g for AB capsule oil. The results demonstrate that there is no significant difference in MP levels between PB and AB raw oils. Comparison between the raw and capsule oils indicated that MP contamination primarily results from the encapsulation process rather than the source of raw material. PP and PET were the predominant MP species detected (83 to 95%) in the factory. It was observed that MPs are detected at a higher frequency as their size decreases. The results of this research provide data for the occurrence of MPs in omega-3 oils based on their sources using reliable methods. It is necessary to acknowledge the limitations of the small number of PB raw oil samples and the lack of directly matched capsule oil and its raw material. Based on the results, the continued development of test methods and further study of other functional foods will be required to address human exposure to MPs and its possible health effects.

## Figures and Tables

**Table 1:** List of omega-3 oil samples.

Sample Type	Oil Source	The Number of Samples
Raw oil	Plant-based (PB)	2
	Animal-based (AB)	12
Capsule oil	Plant-based (PB)	7
	Animal-based (AB)	14

## Data Availability

The original contributions presented in the study are included in the article and Supplementary Materials, further inquiries can be directed to the corresponding authors.

## Supplementary Materials

The following supporting information can be downloaded at https://www.mdpi.com/article/10.3390/foods13101434/s1, Figure S1: PS spheres used for acetone damage test: (a) before treatment; (b) after treatment, red circle shows slightly dissolved surfaced. Figure S2: PE spheres used for recovery test: (a) before addition; (b) recovered PE spheres on filter. Figure S3: Distribution by polymer size of (a) PB raw oil; (b) PB capsule oil; (c) AB raw oil; (d) AB capsule oil. Table S1: Numbers of MPs in omega-3 samples.
