# Supplementary material for: Determination of Microplastics in Omega-3 Oil Supplements
Source: Foods. 2024 May 7;13(10):1434. doi: 10.3390/foods13101434 (PMC11120427; doi:10.3390/foods13101434)
Supplement: Supplementary file 1 [file foods-13-01434-s001.zip › foods-2968854-supplementary.pdf]

Supplementary Materials

Determination of Microplastics in Omega-3 Oil Supplements

Moonhae Kim [1,2,†], Juyang Kim [2,†], Seulah Park [2], Dowoon Kim [2], Jaehak Jung [2,*,‡] and Dongha Cho [1,*,‡]

[1] Department of Bio-Health Convergence, College of Biomedical Science, Kangwon National University, Chuncheon 24341, Republic of Korea; kmhsmsystech@gmail.com (M.K.)

[2] Korea Institute of Analytical Science and Technology, Seoul 04790, Republic of Korea; kjy@kiast.co.kr (J.K.); psa@kiast.co.kr (S.P.); kdw@kiast.co.kr (D.K.)

* Correspondence: jh3370@naver.com (J.J.); chodh@kangwon.ac.kr (D.C.)

† These authors contributed equally to this work.

‡ These authors also contributed equally to this work.

Supplementary Results

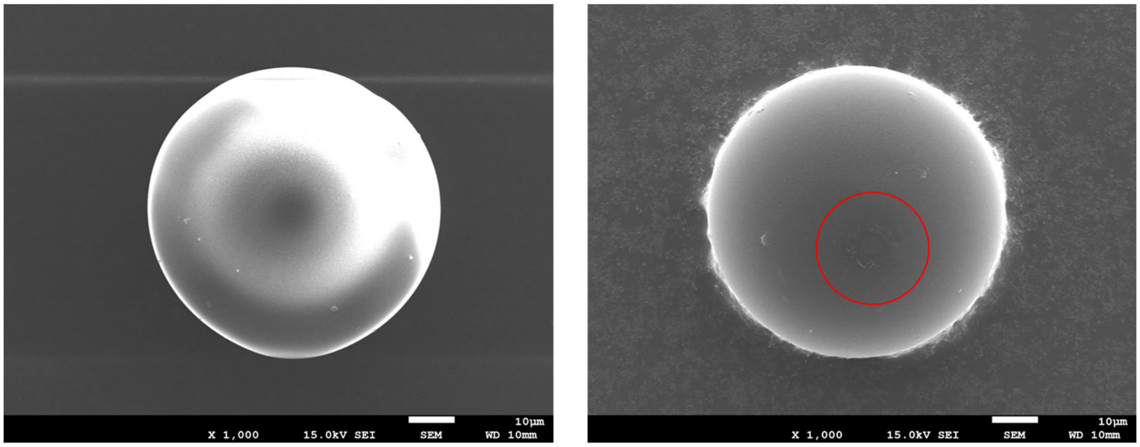

(a) (b)

Figure S1. PS spheres used for acetone damage test: (a)before treatment; (b) after treatment, red circle shows slightly dissolved surfaced.

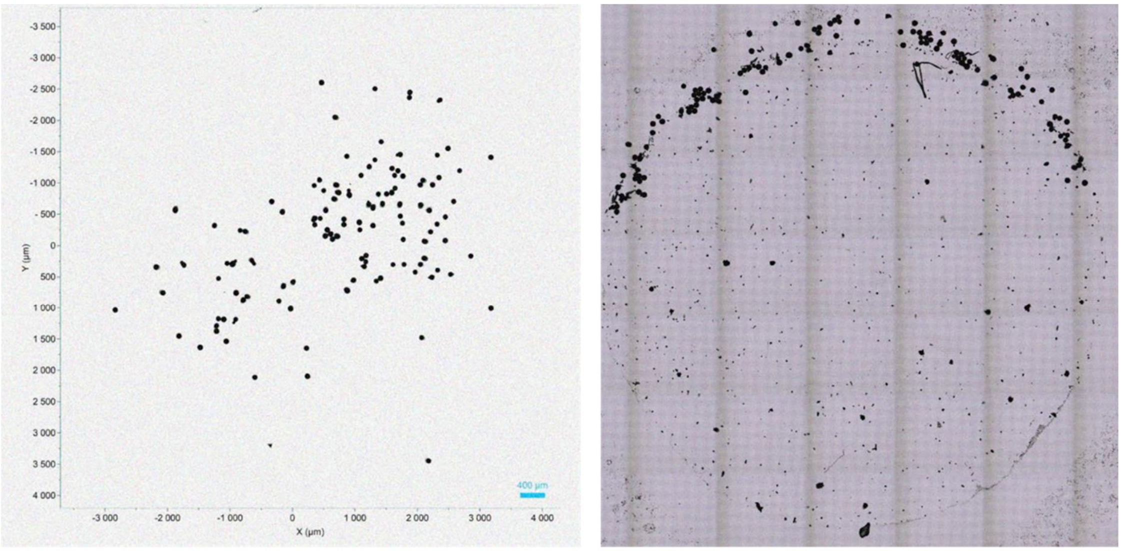

(a) (b)

Figure S2. PE spheres used for recovery test: (a)before addition; (b) recovered PE spheres on filter.

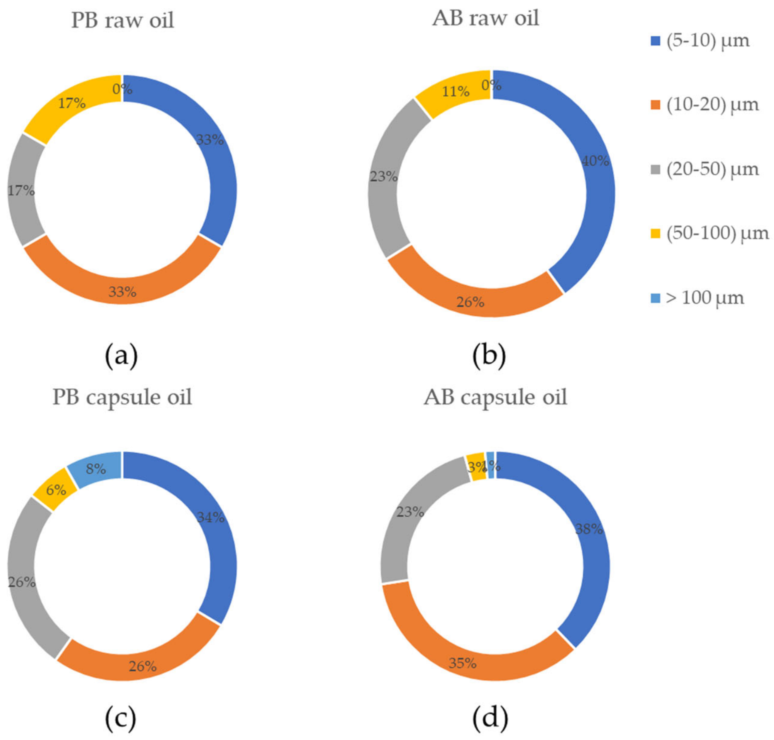

Figure S3. Distribution by polymer size of: (a) PB raw oil; (b) PB capsule oil; (c) AB raw oil; (d) AB capsule oil.

Table S1. Numbers of MPs in omega-3 samples

Sample type	Based oil	Total number of MPs	Composition of MPs	Amount of sample (g)
Raw oil	PB	6	3 PP, 1 PS, 2 PET	2.5
Raw oil	PB	0	N.D.*	2.5
Raw oil	AB	7	7 PP	2.5
Raw oil	AB	6	5 PP, 1 PET	2.5
Raw oil	AB	1	1 PP	2.5
Raw oil	AB	10	8 PP, 1 PS, 1 PET	2.5
Raw oil	AB	2	2 PP	2.5
Raw oil	AB	7	7 PP	2.5
Raw oil	AB	2	2 PP	2.5
Raw oil	AB	2	1 PE, 1 PET	2.5
Raw oil	AB	3	3 PP	2.5
Raw oil	AB	14	14 PP	2.5
Raw oil	AB	8	5 PP, 3 PET	2.5
Raw oil	AB	1	1 PP	2.5
Capsule oil	PB	34	17, PP, 16 PET, 1 PVC	2.9
Capsule oil	PB	7	7 PP	1.4
Capsule oil	PB	4	3 PP, 1 PVC	2.7
Capsule oil	PB	5	1 PE, 4 PET	2.2
Capsule oil	PB	0	N.D.	3.0
Capsule oil	PB	8	4 PP, 1 PE, 3 PET	3.0
Capsule oil	PB	5	1 PP, 4 PET	3.0
Capsule oil	AB	0	N.D.	3.0
Capsule oil	AB	13	8 PP, 3 PET, 2 PMMA	2.1
Capsule oil	AB	9	8 PP, 1 PS	2.1
Capsule oil	AB	11	10 PP, 1 PMMA	1.1
Capsule oil	AB	8	8 PP	1.1
Capsule oil	AB	17	7 PP, 10 PET	2.5
Capsule oil	AB	73	53 PP, 20 PET	2.4
Capsule oil	AB	44	6 PP, 34 PET, 4 PVC	2.8
Capsule oil	AB	52	21 PP, 31 PET	2.7
Capsule oil	AB	18	8 PP, 10 PET	0.8
Capsule oil	AB	34	26 PP, 8 PE	2.2
Capsule oil	AB	0	N.D.	1.1
Capsule oil	AB	8	5 PP, 3 PET	1.1
Capsule oil	AB	3	2 PP, 1 PET	1.0
Raw oil	PB	6	3 PP, 1 PS, 2 PET	2.5

*N.D. : Not Detected.
